# Stafne's Defect with Buccal Cortical Expansion: A Case Report

**DOI:** 10.1155/2010/515931

**Published:** 2010-05-04

**Authors:** Paulo Sérgio Flores Campos, José Aloysio Carvalho Oliveira, Janaina Araújo Dantas, Daniela Pita de Melo, Nilson Pena, Luís Antônio Nogueira Santos, Iêda Margarida Rocha Crusoé-Rebello

**Affiliations:** ^1^Department of Radiology, School of Dentistry, Federal University of Bahia, 40110-150 Bahia, Brazil; ^2^Clinical Dentist, Serviço Odontológico de Diagnóstico Computadorizado (SODIC), Department of Radiology, 49.015-130 Aracaju, Sergipe, Brazil; ^3^Department of Radiology, Piracicaba Dental School, University of Campinas, 13414-903 São Paulo, Brazil; ^4^Fundação de Amparo à Pesquisa do Estado da Bahia, 40210-720 Bahia, Brazil

## Abstract

A rare case of Stafne's bone cavity, type III-G, is reported in a 49-year-old male patient who had been referred to a private clinic for a routine evaluation. The final diagnosis was based on computed tomography. Scintigraphy played a fundamental role in determining the most likely etiology.

## 1. Introduction

Stafne [1] first described lingual bone cavities near the mandibular angle and, based on data collected from his 34 patients (35 bone cavities), established that this condition involves mainly male patients between 40 and 50 years of age, is evenly distributed on both sides, and does not cause symptoms. The shape of a lingual bone cavity can be round or oval, and it varies from 1 to 3 cm in diameter. When the cavity is oval, the major axis is parallel to the mandibular border, and if it is wider than 3 cm, there may be interference in the continuity of the mandibular border, which can be felt by means of palpation. Stafne's bone defects (SBDs) occur below the mandibular canal, in an anterior position in relation to the angle of the mandible, at the level of the third molar. Radiographically, the cortical outline of the bone defect is denser and thicker than that of odontogenic cysts. Five of the 35 bone cavities were followed up over a period of more than 11 years and no change in size was noted.

Using computed tomography (CT) images, Ariji et al. [2] classified SBDs according to the depth and content of the cavities. According to depth, the bone defects were classified as follows.

- *Type I*: Cavity depth is limited to the medullar portion of the mandible.
- *Type II*: Cavity depth reaches the buccal cortex of the mandible but does not cause its expansion.
- *Type III*: Cavity depth reaches the buccal cortex of the mandible and causes its expansion.

According to content, they were classified as follows.

- *Type F*: Cavity is filled with fat.
- *Type S*: Cavity is filled with soft tissue (lymphonode, vessel, conjunctive tissue, etc.).
- *Type G*: Cavity is filled with part of the submandibular gland.

The aim of this work is to report on the rarest occurrence of this type of bone defect: SBD type III-G.

## 2. Case Report

**Figure 1:**
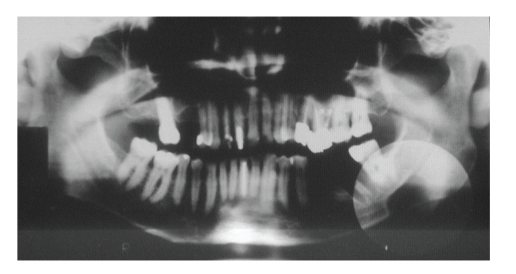
Panoramic radiography showing the radiolucent area.

A 49-year-old asymptomatic male patient was referred to a private clinic in order to undergo routine panoramic radiography. Results showed an oval-shaped, radiolucent area of cystic aspect and regular, well-defined cortical outline; its longest axis was placed horizontally in the left hemimandible. This lithic area, located under the lower left third molar, was anterior to the mandibular angle, and reached the border of the mandible, which showed thinner than normal width due to the presence of the bone defect. The apparent unity of the upper contour of the lesion and the upper wall of the mandibular canal gave them a thicker than normal appearance. The fact that the lower wall of the mandibular canal was visible within the radiolucent area showed that there could be a neighboring relationship, but not an involvement, of the inferior alveolar nerve (Figure 1).

**Figure 2:**
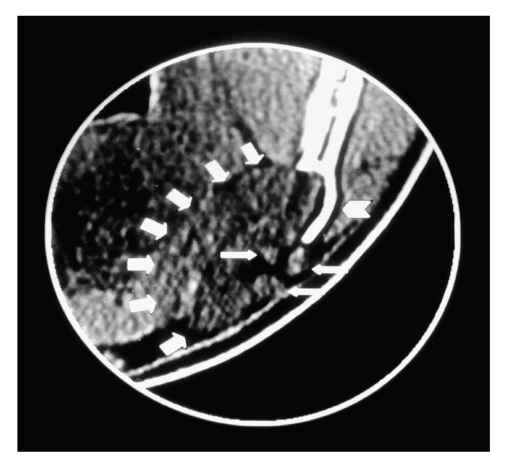
CT image:soft-tissue window and coronal view, in which the expansion of the mandibular buccal cortex (arrow head), the submandibular gland (large arrows), and some lymph nodes (small arrows) can be seen.

**Figure 3:**
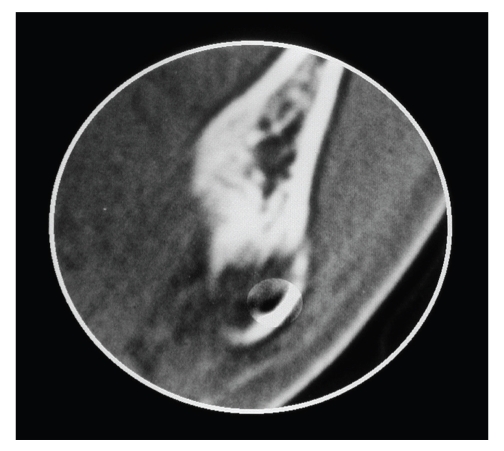
CT image:bone window and coronal view, in which the buccal location of the mandibular canal can be seen.

As a hard buccal protuberance was perceived on palpation, a CT was performed. The coronal view in the CT scan showed that there had been a distention of the mandibular buccal cortex and that glandular tissue had been spreading into the bone defect. A further coronal view revealed the buccal location of the mandibular canal, which was preserved (Figures 2 and 3).

**Figure 4:**
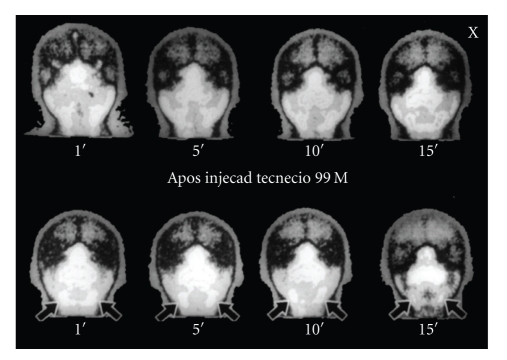
Scintigraphy revealing hyperretention of the radionuclide.

A previous scintigraphy revealed an inflammatory condition associated with an obstructive process involving the submandibular glands, particularly on the left side (Figure 4).

## 3. Discussion

The etiology of SBD was suggested by Stafne [1] to arise as early as the fetal period by failure of normal deposition of bone to fill the cavity formed by regressive changes of the lowest portion of condylar cartilage. Choukas and Toto [3] did not rule out a congenital origin, but advocated that entrapment of the superior lobe of the submandibular gland during mandible development would determine the bone defect formation. They speculated that SBD may be the result of an erosive process caused by the superior lobe of the hypertrophic gland. A bone defect formation was documented by Tolman and Stafne [4] confirming that SBD may also have a developmental origin.

Recently, an extensive literature review [5] affirmed that all SBD variants (anterior:related to the sublingual gland, posterior:related to the submandibular gland, and of the ascending ramus:related to the parotid gland) are the result of an erosive process caused by pressure of hypertrophic/hyperplastic submandibular glands on the bone surface. However, the area of medial pterygoid muscle attachment was included as a site of SBD (posterior variant) presentation in Philipsen et al.'s study [5], where contact between the gland and mandible surface is improbable.

Minowa et al. [6] did not consider it reasonable that the gland exerts pressure and cavitation on the medial aspect of the mandible and speculated that SBD may have its origin in a benign lipoma or be the result of bone resorption due to an acquired vascular lesion. In addition, they refute the embryologic and congenital origin since SBD does not occur in children under 10 years of age.

In a report on a mandibular ramus-related Stafne's bone cavity [7], Campos et al. showed that there was no contact between the parotid gland and the bone surface at the site. Therefore, SBD may also be the result of a focal failure during intramembranous ossification of the mandible [7].

More recently, Shimizu et al. [8] refuted the vascular origin; SBD was assumed to be a gland-related condition, and dislocation of the submandibular gland was proposed as the cause of its occurrence.

This particular case clearly shows the relationship between the cavity and its corresponding submandibular gland. Due to a chronic inflammatory process detected in the scintigraphy, the gland seems hypertrophied and capable of exerting enough pressure to cause bone resorption. Moreover, we consider this pressure sufficient to cause distension of the buccal cortex. Thus, we agree with Campos [9] when he says that (1) submandibular gland hyperplasia/hypertrophy is the chief etiologic factor for the vast majority of cases of SBD, posterior variant, in the area free from medial pterygoid muscle attachment (2) a few cases of SBD, posterior variant, mostly in the area of medial pterygoid muscle attachment, are defects of embryologic origin and (3) vascular alterations have a contributory but not the main role in SBD formation.
